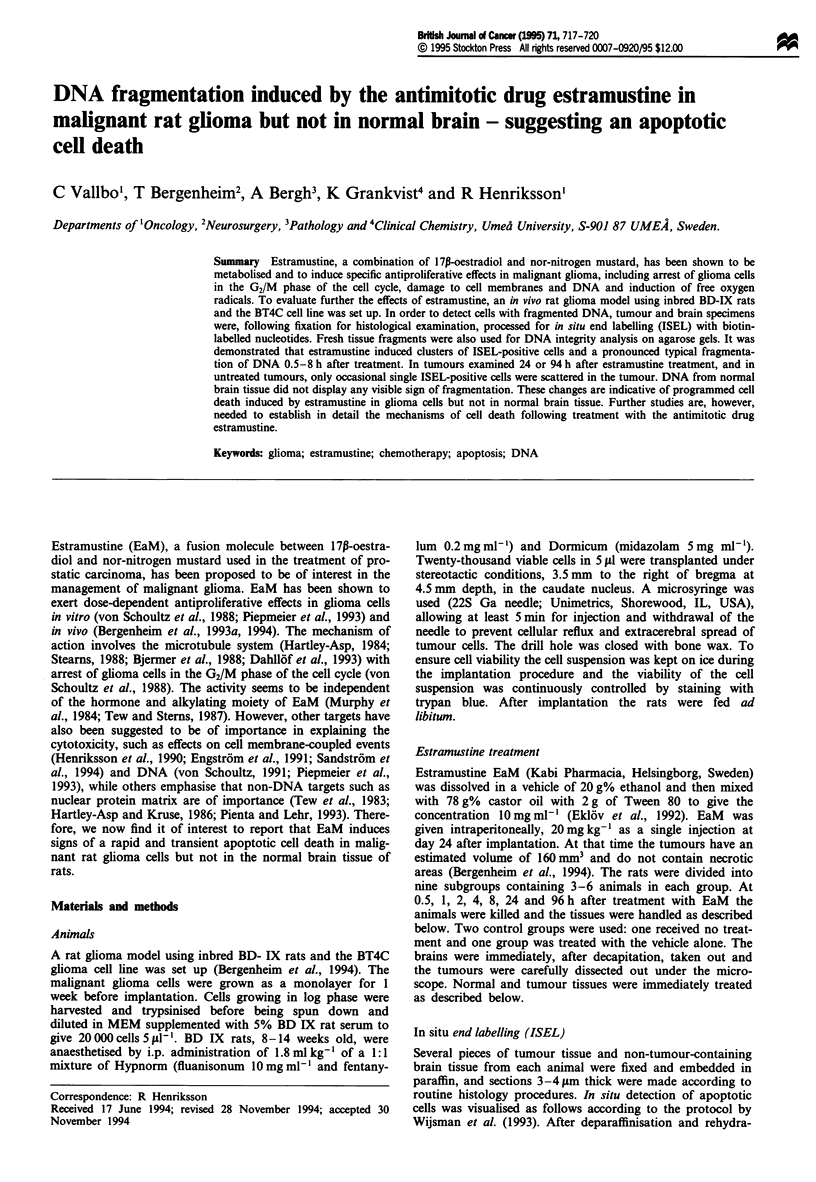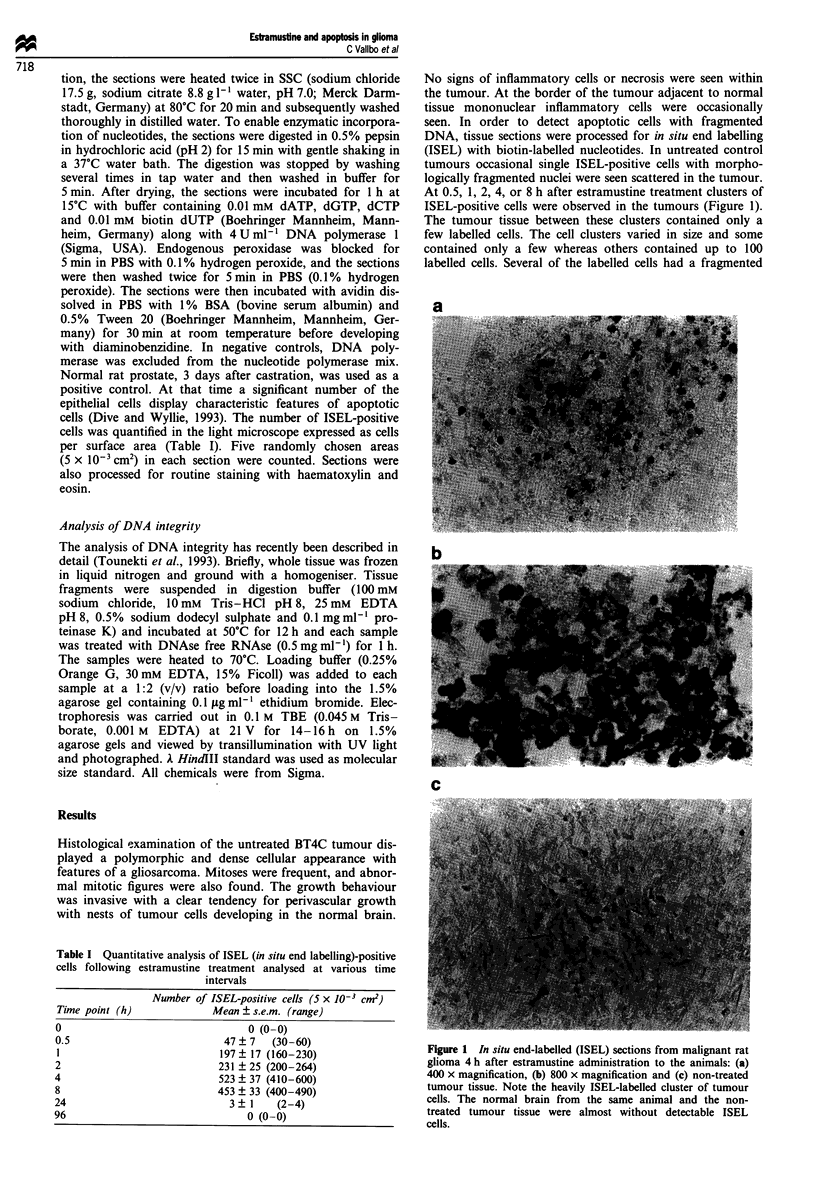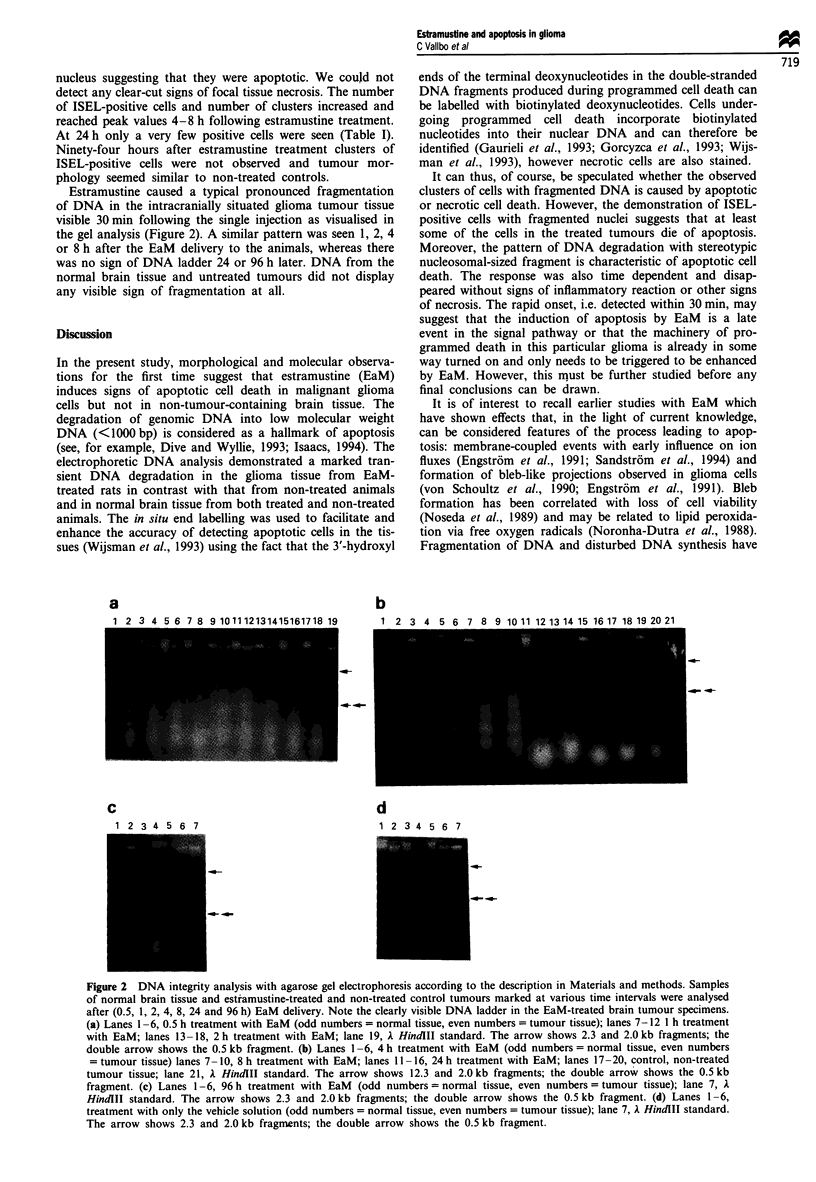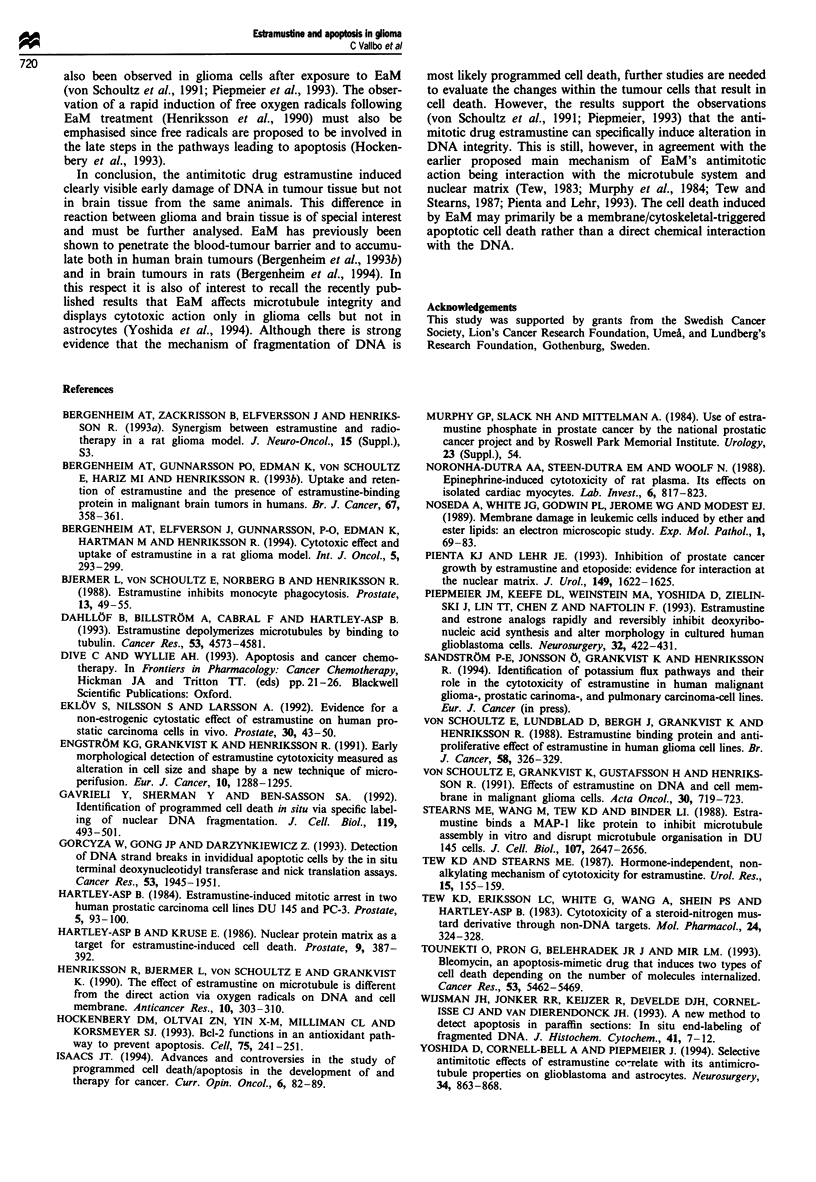# DNA fragmentation induced by the antimitotic drug estramustine in malignant rat glioma but not in normal brain--suggesting an apoptotic cell death.

**DOI:** 10.1038/bjc.1995.140

**Published:** 1995-04

**Authors:** C. Vallbo, T. Bergenheim, A. Bergh, K. Grankvist, R. Henriksson

**Affiliations:** Department of Oncology, Umeå University, Sweden.

## Abstract

**Images:**


British Journal of Cancer (1995) 71, 717–720

© 1995 Stockton Press All rights reserved 0007-0920/95 $12.00

### DNA fragmentation induced by the antimitotic drug estramustine in malignant rat glioma but not in normal brain – suggesting an apoptotic cell death

C Vallbo[1], T Bergenheim[2], A Bergh[3], K Grankvist[4] and R Henriksson[1]

*Departments of [1]Oncology, [2]Neurosurgery, [3]Pathology and [4]Clinical Chemistry, Umeå University, S-901 87 UMEÅ, Sweden.*

**Summary** Estramustine, a combination of 17β-oestradiol and nor-nitrogen mustard, has been shown to be metabolised and to induce specific antiproliferative effects in malignant glioma, including arrest of glioma cells in the $G_2/M$ phase of the cell cycle, damage to cell membranes and DNA and induction of free oxygen radicals. To evaluate further the effects of estramustine, an *in vivo* rat glioma model using inbred BD-IX rats and the BT4C cell line was set up. In order to detect cells with fragmented DNA, tumour and brain specimens were, following fixation for histological examination, processed for *in situ* end labelling (ISEL) with biotin-labelled nucleotides. Fresh tissue fragments were also used for DNA integrity analysis on agarose gels. It was demonstrated that estramustine induced clusters of ISEL-positive cells and a pronounced typical fragmentation of DNA 0.5–8 h after treatment. In tumours examined 24 or 94 h after estramustine treatment, and in untreated tumours, only occasional single ISEL-positive cells were scattered in the tumour. DNA from normal brain tissue did not display any visible sign of fragmentation. These changes are indicative of programmed cell death induced by estramustine in glioma cells but not in normal brain tissue. Further studies are, however, needed to establish in detail the mechanisms of cell death following treatment with the antimitotic drug estramustine.

**Keywords:** glioma; estramustine; chemotherapy; apoptosis; DNA

Estramustine (EaM), a fusion molecule between 17β-oestradiol and nor-nitrogen mustard used in the treatment of prostatic carcinoma, has been proposed to be of interest in the management of malignant glioma. EaM has been shown to exert dose-dependent antiproliferative effects in glioma cells *in vitro* (von Schoultz *et al.*, 1988; Piepmeier *et al.*, 1993) and *in vivo* (Bergenheim *et al.*, 1993*a*, 1994). The mechanism of action involves the microtubule system (Hartley-Asp, 1984; Stearns, 1988; Bjermer *et al.*, 1988; Dahllöf *et al.*, 1993) with arrest of glioma cells in the $G_2/M$ phase of the cell cycle (von Schoultz *et al.*, 1988). The activity seems to be independent of the hormone and alkylating moiety of EaM (Murphy *et al.*, 1984; Tew and Sterns, 1987). However, other targets have also been suggested to be of importance in explaining the cytotoxicity, such as effects on cell membrane-coupled events (Henriksson *et al.*, 1990; Engström *et al.*, 1991; Sandström *et al.*, 1994) and DNA (von Schoultz, 1991; Piepmeier *et al.*, 1993), while others emphasise that non-DNA targets such as nuclear protein matrix are of importance (Tew *et al.*, 1983; Hartley-Asp and Kruse, 1986; Pienta and Lehr, 1993). Therefore, we now find it of interest to report that EaM induces signs of a rapid and transient apoptotic cell death in malignant rat glioma cells but not in the normal brain tissue of rats.

### Materials and methods

### Animals

A rat glioma model using inbred BD- IX rats and the BT4C glioma cell line was set up (Bergenheim *et al.*, 1994). The malignant glioma cells were grown as a monolayer for 1 week before implantation. Cells growing in log phase were harvested and trypsinised before being spun down and diluted in MEM supplemented with 5% BD IX rat serum to give 20 000 cells 5 $\mu l^{-1}$. BD IX rats, 8–14 weeks old, were anaesthetised by i.p. administration of 1.8 ml $kg^{-1}$ of a 1:1 mixture of Hypnorm (fluanisonum 10 mg $ml^{-1}$ and fentanylum 0.2 mg $ml^{-1}$) and Dormicum (midazolam 5 mg $ml^{-1}$). Twenty-thousand viable cells in 5 µl were transplanted under stereotactic conditions, 3.5 mm to the right of bregma at 4.5 mm depth, in the caudate nucleus. A microsyringe was used (22S Ga needle; Unimetrics, Shorewood, IL, USA), allowing at least 5 min for injection and withdrawal of the needle to prevent cellular reflux and extracerebral spread of tumour cells. The drill hole was closed with bone wax. To ensure cell viability the cell suspension was kept on ice during the implantation procedure and the viability of the cell suspension was continuously controlled by staining with trypan blue. After implantation the rats were fed *ad libitum.*

### Estramustine treatment

Estramustine EaM (Kabi Pharmacia, Helsingborg, Sweden) was dissolved in a vehicle of 20 g% ethanol and then mixed with 78 g% castor oil with 2 g of Tween 80 to give the concentration 10 mg $ml^{-1}$ (Eklöv *et al.*, 1992). EaM was given intraperitoneally, 20 mg $kg^{-1}$ as a single injection at day 24 after implantation. At that time the tumours have an estimated volume of 160 $mm^3$ and do not contain necrotic areas (Bergenheim *et al.*, 1994). The rats were divided into nine subgroups containing 3–6 animals in each group. At 0.5, 1, 2, 4, 8, 24 and 96 h after treatment with EaM the animals were killed and the tissues were handled as described below. Two control groups were used: one received no treatment and one group was treated with the vehicle alone. The brains were immediately, after decapitation, taken out and the tumours were carefully dissected out under the microscope. Normal and tumour tissues were immediately treated as described below.

### In situ *end labelling (ISEL)*

Several pieces of tumour tissue and non-tumour-containing brain tissue from each animal were fixed and embedded in paraffin, and sections 3–4 µm thick were made according to routine histology procedures. *In situ* detection of apoptotic cells was visualised as follows according to the protocol by Wijsman *et al.* (1993). After deparaffinisation and rehydra-

Correspondence: R Henriksson
Received 17 June 1994; revised 28 November 1994; accepted 30 November 1994

tion, the sections were heated twice in SSC (sodium chloride 17.5 g, sodium citrate 8.8 g $l^{-1}$ water, pH 7.0; Merck Darmstadt, Germany) at 80°C for 20 min and subsequently washed thoroughly in distilled water. To enable enzymatic incorporation of nucleotides, the sections were digested in 0.5% pepsin in hydrochloric acid (pH 2) for 15 min with gentle shaking in a 37°C water bath. The digestion was stopped by washing several times in tap water and then washed in buffer for 5 min. After drying, the sections were incubated for 1 h at 15°C with buffer containing 0.01 mM dATP, dGTP, dCTP and 0.01 mM biotin dUTP (Boehringer Mannheim, Mannheim, Germany) along with 4 U $ml^{-1}$ DNA polymerase 1 (Sigma, USA). Endogenous peroxidase was blocked for 5 min in PBS with 0.1% hydrogen peroxide, and the sections were then washed twice for 5 min in PBS (0.1% hydrogen peroxide). The sections were then incubated with avidin dissolved in PBS with 1% BSA (bovine serum albumin) and 0.5% Tween 20 (Boehringer Mannheim, Mannheim, Germany) for 30 min at room temperature before developing with diaminobenzidine. In negative controls, DNA polymerase was excluded from the nucleotide polymerase mix. Normal rat prostate, 3 days after castration, was used as a positive control. At that time a significant number of the epithelial cells display characteristic features of apoptotic cells (Dive and Wyllie, 1993). The number of ISEL-positive cells was quantified in the light microscope expressed as cells per surface area (Table I). Five randomly chosen areas ($5 \times 10^{-3}$ $cm^2$) in each section were counted. Sections were also processed for routine staining with haematoxylin and eosin.

### *Analysis of DNA integrity*

The analysis of DNA integrity has recently been described in detail (Tounekti *et al.*, 1993). Briefly, whole tissue was frozen in liquid nitrogen and ground with a homogeniser. Tissue fragments were suspended in digestion buffer (100 mM sodium chloride, 10 mM Tris-HCl pH 8, 25 mM EDTA pH 8, 0.5% sodium dodecyl sulphate and 0.1 mg $ml^{-1}$ proteinase K) and incubated at 50°C for 12 h and each sample was treated with DNAse free RNAse (0.5 mg $ml^{-1}$) for 1 h. The samples were heated to 70°C. Loading buffer (0.25% Orange G, 30 mM EDTA, 15% Ficoll) was added to each sample at a 1:2 (v/v) ratio before loading into the 1.5% agarose gel containing 0.1 μg $ml^{-1}$ ethidium bromide. Electrophoresis was carried out in 0.1 M TBE (0.045 M Tris-borate, 0.001 M EDTA) at 21 V for 14-16 h on 1.5% agarose gels and viewed by transillumination with UV light and photographed. λ *Hind*III standard was used as molecular size standard. All chemicals were from Sigma.

### Results

Histological examination of the untreated BT4C tumour displayed a polymorphic and dense cellular appearance with features of a gliosarcoma. Mitoses were frequent, and abnormal mitotic figures were also found. The growth behaviour was invasive with a clear tendency for perivascular growth with nests of tumour cells developing in the normal brain.

**Table I** Quantitative analysis of ISEL (*in situ* end labelling)-positive cells following estramustine treatment analysed at various time intervals

| *Time point (h)* | *Number of ISEL-positive cells (5 × 10⁻³ cm²)* *Mean ± s.e.m. (range)* |
|---|---|
| 0 | 0 (0-0) |
| 0.5 | 47 ± 7 (30-60) |
| 1 | 197 ± 17 (160-230) |
| 2 | 231 ± 25 (200-264) |
| 4 | 523 ± 37 (410-600) |
| 8 | 453 ± 33 (400-490) |
| 24 | 3 ± 1 (2-4) |
| 96 | 0 (0-0) |

No signs of inflammatory cells or necrosis were seen within the tumour. At the border of the tumour adjacent to normal tissue mononuclear inflammatory cells were occasionally seen. In order to detect apoptotic cells with fragmented DNA, tissue sections were processed for *in situ* end labelling (ISEL) with biotin-labelled nucleotides. In untreated control tumours occasional single ISEL-positive cells with morphologically fragmented nuclei were seen scattered in the tumour. At 0.5, 1, 2, 4, or 8 h after estramustine treatment clusters of ISEL-positive cells were observed in the tumours (Figure 1). The tumour tissue between these clusters contained only a few labelled cells. The cell clusters varied in size and some contained only a few whereas others contained up to 100 labelled cells. Several of the labelled cells had a fragmented

**Figure 1** *In situ* end-labelled (ISEL) sections from malignant rat glioma 4 h after estramustine administration to the animals: (**a**) 400 × magnification, (**b**) 800 × magnification and (**c**) non-treated tumour tissue. Note the heavily ISEL-labelled cluster of tumour cells. The normal brain from the same animal and the non-treated tumour tissue were almost without detectable ISEL cells.

nucleus suggesting that they were apoptotic. We could not detect any clear-cut signs of focal tissue necrosis. The number of ISEL-positive cells and number of clusters increased and reached peak values 4–8 h following estramustine treatment. At 24 h only a very few positive cells were seen (Table I). Ninety-four hours after estramustine treatment clusters of ISEL-positive cells were not observed and tumour morphology seemed similar to non-treated controls.

Estramustine caused a typical pronounced fragmentation of DNA in the intracranially situated glioma tumour tissue visible 30 min following the single injection as visualised in the gel analysis (Figure 2). A similar pattern was seen 1, 2, 4 or 8 h after the EaM delivery to the animals, whereas there was no sign of DNA ladder 24 or 96 h later. DNA from the normal brain tissue and untreated tumours did not display any visible sign of fragmentation at all.

### Discussion

In the present study, morphological and molecular observations for the first time suggest that estramustine (EaM) induces signs of apoptotic cell death in malignant glioma cells but not in non-tumour-containing brain tissue. The degradation of genomic DNA into low molecular weight DNA (<1000 bp) is considered as a hallmark of apoptosis (see, for example, Dive and Wyllie, 1993; Isaacs, 1994). The electrophoretic DNA analysis demonstrated a marked transient DNA degradation in the glioma tissue from EaM-treated rats in contrast with that from non-treated animals and in normal brain tissue from both treated and non-treated animals. The *in situ* end labelling was used to facilitate and enhance the accuracy of detecting apoptotic cells in the tissues (Wijsman *et al.*, 1993) using the fact that the 3′-hydroxyl ends of the terminal deoxynucleotides in the double-stranded DNA fragments produced during programmed cell death can be labelled with biotinylated deoxynucleotides. Cells undergoing programmed cell death incorporate biotinylated nucleotides into their nuclear DNA and can therefore be identified (Gaurieli *et al.*, 1993; Gorcyzca *et al.*, 1993; Wijsman *et al.*, 1993), however necrotic cells are also stained.

It can thus, of course, be speculated whether the observed clusters of cells with fragmented DNA is caused by apoptotic or necrotic cell death. However, the demonstration of ISEL-positive cells with fragmented nuclei suggests that at least some of the cells in the treated tumours die of apoptosis. Moreover, the pattern of DNA degradation with stereotypic nucleosomal-sized fragment is characteristic of apoptotic cell death. The response was also time dependent and disappeared without signs of inflammatory reaction or other signs of necrosis. The rapid onset, i.e. detected within 30 min, may suggest that the induction of apoptosis by EaM is a late event in the signal pathway or that the machinery of programmed death in this particular glioma is already in some way turned on and only needs to be triggered to be enhanced by EaM. However, this must be further studied before any final conclusions can be drawn.

It is of interest to recall earlier studies with EaM which have shown effects that, in the light of current knowledge, can be considered features of the process leading to apoptosis: membrane-coupled events with early influence on ion fluxes (Engström *et al.*, 1991; Sandström *et al.*, 1994) and formation of bleb-like projections observed in glioma cells (von Schoultz *et al.*, 1990; Engström *et al.*, 1991). Bleb formation has been correlated with loss of cell viability (Noseda *et al.*, 1989) and may be related to lipid peroxidation via free oxygen radicals (Noronha-Dutra *et al.*, 1988). Fragmentation of DNA and disturbed DNA synthesis have

**Figure 2** DNA integrity analysis with agarose gel electrophoresis according to the description in Materials and methods. Samples of normal brain tissue and estramustine-treated and non-treated control tumours marked at various time intervals were analysed after (0.5, 1, 2, 4, 8, 24 and 96 h) EaM delivery. Note the clearly visible DNA ladder in the EaM-treated brain tumour specimens. (**a**) Lanes 1–6, 0.5 h treatment with EaM (odd numbers = normal tissue, even numbers = tumour tissue); lanes 7–12 1 h treatment with EaM; lanes 13–18, 2 h treatment with EaM; lane 19, λ *Hind*III standard. The arrow shows 2.3 and 2.0 kb fragments; the double arrow shows the 0.5 kb fragment. (**b**) Lanes 1–6, 4 h treatment with EaM (odd numbers = normal tissue, even numbers = tumour tissue) lanes 7–10, 8 h treatment with EaM; lanes 11–16, 24 h treatment with EaM; lanes 17–20, control, non-treated tumour tissue; lane 21, λ *Hind*III standard. The arrow shows 12.3 and 2.0 kb fragments; the double arrow shows the 0.5 kb fragment. (**c**) Lanes 1–6, 96 h treatment with EaM (odd numbers = normal tissue, even numbers = tumour tissue); lane 7, λ *Hind*III standard. The arrow shows 2.3 and 2.0 kb fragments; the double arrow shows the 0.5 kb fragment. (**d**) Lanes 1–6, treatment with only the vehicle solution (odd numbers = normal tissue, even numbers = tumour tissue); lane 7, λ *Hind*III standard. The arrow shows 2.3 and 2.0 kb fragments; the double arrow shows the 0.5 kb fragment.

also been observed in glioma cells after exposure to EaM (von Schoultz et al., 1991; Piepmeier et al., 1993). The observation of a rapid induction of free oxygen radicals following EaM treatment (Henriksson et al., 1990) must also be emphasised since free radicals are proposed to be involved in the late steps in the pathways leading to apoptosis (Hockenbery et al., 1993).

In conclusion, the antimitotic drug estramustine induced clearly visible early damage of DNA in tumour tissue but not in brain tissue from the same animals. This difference in reaction between glioma and brain tissue is of special interest and must be further analysed. EaM has previously been shown to penetrate the blood-tumour barrier and to accumulate both in human brain tumours (Bergenheim et al., 1993b) and in brain tumours in rats (Bergenheim et al., 1994). In this respect it is also of interest to recall the recently published results that EaM affects microtubule integrity and displays cytotoxic action only in glioma cells but not in astrocytes (Yoshida et al., 1994). Although there is strong evidence that the mechanism of fragmentation of DNA is most likely programmed cell death, further studies are needed to evaluate the changes within the tumour cells that result in cell death. However, the results support the observations (von Schoultz et al., 1991; Piepmeier, 1993) that the antimitotic drug estramustine can specifically induce alteration in DNA integrity. This is still, however, in agreement with the earlier proposed main mechanism of EaM's antimitotic action being interaction with the microtubule system and nuclear matrix (Tew, 1983; Murphy et al., 1984; Tew and Stearns, 1987; Pienta and Lehr, 1993). The cell death induced by EaM may primarily be a membrane/cytoskeletal-triggered apoptotic cell death rather than a direct chemical interaction with the DNA.

**Acknowledgements**
This study was supported by grants from the Swedish Cancer Society, Lion's Cancer Research Foundation, Umeå, and Lundberg's Research Foundation, Gothenburg, Sweden.

**References**

BERGENHEIM AT, ZACKRISSON B, ELFVERSSON J AND HENRIKSSON R. (1993a). Synergism between estramustine and radiotherapy in a rat glioma model. *J. Neuro-Oncol.*, **15** (Suppl.), S3.

BERGENHEIM AT, GUNNARSSON PO, EDMAN K, VON SCHOULTZ E, HARIZ MI AND HENRIKSSON R. (1993b). Uptake and retention of estramustine and the presence of estramustine-binding protein in malignant brain tumors in humans. *Br. J. Cancer*, **67**, 358–361.

BERGENHEIM AT, ELFVERSON J, GUNNARSSON, P-O, EDMAN K, HARTMAN M AND HENRIKSSON R. (1994). Cytotoxic effect and uptake of estramustine in a rat glioma model. *Int. J. Oncol.*, **5**, 293–299.

BJERMER L, VON SCHOULTZ E, NORBERG B AND HENRIKSSON R. (1988). Estramustine inhibits monocyte phagocytosis. *Prostate*, **13**, 49–55.

DAHLLÖF B, BILLSTRÖM A, CABRAL F AND HARTLEY-ASP B. (1993). Estramustine depolymerizes microtubules by binding to tubulin. *Cancer Res.*, **53**, 4573–4581.

DIVE C AND WYLLIE AH. (1993). Apoptosis and cancer chemotherapy. In *Frontiers in Pharmacology: Cancer Chemotherapy*, Hickman JA and Tritton TT. (eds) pp. 21–26. Blackwell Scientific Publications: Oxford.

EKLÖV S, NILSSON S AND LARSSON A. (1992). Evidence for a non-estrogenic cytostatic effect of estramustine on human prostatic carcinoma cells in vivo. *Prostate*, **30**, 43–50.

ENGSTRÖM KG, GRANKVIST K AND HENRIKSSON R. (1991). Early morphological detection of estramustine cytotoxicity measured as alteration in cell size and shape by a new technique of microperifusion. *Eur. J. Cancer*, **10**, 1288–1295.

GAVRIELI Y, SHERMAN Y AND BEN-SASSON SA. (1992). Identification of programmed cell death *in situ* via specific labeling of nuclear DNA fragmentation. *J. Cell. Biol.*, **119**, 493–501.

GORCYZA W, GONG JP AND DARZYNKIEWICZ Z. (1993). Detection of DNA strand breaks in invididual apoptotic cells by the in situ terminal deoxynucleotidyl transferase and nick translation assays. *Cancer Res.*, **53**, 1945–1951.

HARTLEY-ASP B. (1984). Estramustine-induced mitotic arrest in two human prostatic carcinoma cell lines DU 145 and PC-3. *Prostate*, **5**, 93–100.

HARTLEY-ASP B AND KRUSE E. (1986). Nuclear protein matrix as a target for estramustine-induced cell death. *Prostate*, **9**, 387–392.

HENRIKSSON R, BJERMER L, VON SCHOULTZ E AND GRANKVIST K. (1990). The effect of estramustine on microtubule is different from the direct action via oxygen radicals on DNA and cell membrane. *Anticancer Res.*, **10**, 303–310.

HOCKENBERY DM, OLTVAI ZN, YIN X-M, MILLIMAN CL AND KORSMEYER SJ. (1993). Bcl-2 functions in an antioxidant pathway to prevent apoptosis. *Cell*, **75**, 241–251.

ISAACS JT. (1994). Advances and controversies in the study of programmed cell death/apoptosis in the development of and therapy for cancer. *Curr. Opin. Oncol.*, **6**, 82–89.

MURPHY GP, SLACK NH AND MITTELMAN A. (1984). Use of estramustine phosphate in prostate cancer by the national prostatic cancer project and by Roswell Park Memorial Institute. *Urology*, **23** (Suppl.), 54.

NORONHA-DUTRA AA, STEEN-DUTRA EM AND WOOLF N. (1988). Epinephrine-induced cytotoxicity of rat plasma. Its effects on isolated cardiac myocytes. *Lab. Invest.*, **6**, 817–823.

NOSEDA A, WHITE JG, GODWIN PL, JEROME WG AND MODEST EJ. (1989). Membrane damage in leukemic cells induced by ether and ester lipids: an electron microscopic study. *Exp. Mol. Pathol.*, **1**, 69–83.

PIENTA KJ AND LEHR JE. (1993). Inhibition of prostate cancer growth by estramustine and etoposide: evidence for interaction at the nuclear matrix. *J. Urol.*, **149**, 1622–1625.

PIEPMEIER JM, KEEFE DL, WEINSTEIN MA, YOSHIDA D, ZIELINSKI J, LIN TT, CHEN Z AND NAFTOLIN F. (1993). Estramustine and estrone analogs rapidly and reversibly inhibit deoxyribonucleic acid synthesis and alter morphology in cultured human glioblastoma cells. *Neurosurgery*, **32**, 422–431.

SANDSTRÖM P-E, JONSSON Ö, GRANKVIST K AND HENRIKSSON R. (1994). Identification of potassium flux pathways and their role in the cytotoxicity of estramustine in human malignant glioma-, prostatic carinoma-, and pulmonary carcinoma-cell lines. *Eur. J. Cancer* (in press).

VON SCHOULTZ E, LUNDBLAD D, BERGH J, GRANKVIST K AND HENRIKSSON R. (1988). Estramustine binding protein and antiproliferative effect of estramustine in human glioma cell lines. *Br. J. Cancer*, **58**, 326–329.

VON SCHOULTZ E, GRANKVIST K, GUSTAFSSON H AND HENRIKSSON R. (1991). Effects of estramustine on DNA and cell membrane in malignant glioma cells. *Acta Oncol.*, **30**, 719–723.

STEARNS ME, WANG M, TEW KD AND BINDER LI. (1988). Estramustine binds a MAP-1 like protein to inhibit microtubule assembly in vitro and disrupt microtubule organisation in DU 145 cells. *J. Cell. Biol.*, **107**, 2647–2656.

TEW KD AND STEARNS ME. (1987). Hormone-independent, non-alkylating mechanism of cytotoxicity for estramustine. *Urol. Res.*, **15**, 155–159.

TEW KD, ERIKSSON LC, WHITE G, WANG A, SHEIN PS AND HARTLEY-ASP B. (1983). Cytotoxicity of a steroid-nitrogen mustard derivative through non-DNA targets. *Mol. Pharmacol.*, **24**, 324–328.

TOUNEKTI O, PRON G, BELEHRADEK JR J AND MIR LM. (1993). Bleomycin, an apoptosis-mimetic drug that induces two types of cell death depending on the number of molecules internalized. *Cancer Res.*, **53**, 5462–5469.

WIJSMAN JH, JONKER RR, KEIJZER R, DEVELDE DJH, CORNELISSE CJ AND VAN DIERENDONCK JH. (1993). A new method to detect apoptosis in paraffin sections: In situ end-labeling of fragmented DNA. *J. Histochem. Cytochem.*, **41**, 7–12.

YOSHIDA D, CORNELL-BELL A AND PIEPMEIER J. (1994). Selective antimitotic effects of estramustine correlate with its antimicrotubule properties on glioblastoma and astrocytes. *Neurosurgery*, **34**, 863–868.